# Supplementary material for: Continuum of community engagement to ensure access to health care in conflict-affected areas in South Sudan and Central African Republic (CAR): Lessons from the Red Cross
Source: PLOS Glob Public Health. 2026 Feb 10;6(2):e0005794. doi: 10.1371/journal.pgph.0005794 (PMC12890090; doi:10.1371/journal.pgph.0005794)
Supplement: Attachment — Submitted filename: Response to editor-10 Nov.docx [file pgph.0005794.s002.docx]

^Response to editor^

^Thank you for your review and feedback:^

^Please see the required information as follow.^

1. Funding

The funding for this work was provided through the APiH project financially supported by CRC and ICRC. Please see on page 17

2. Competing interests: There is no competing interest among the authors. Please see on page 16
3. Availability of data and materials: We have included most of the required data for this manuscript. Due to ethical considerations and the involvement of human research participants, we are unable to share the full dataset publicly. However, additional data supporting the findings of this study can be made available upon reasonable request. Please contact [@Faiza Rab](mailto:faiza.rab@redcross.ca); Please see on page 16
4. We restate Background to Introduction
5. Now we have attached Figure 1: as a separate document
